# A Novel Inovirus Reprograms Metabolism and Motility of Marine *Alteromonas*

**DOI:** 10.1128/spectrum.03388-22

**Published:** 2022-10-27

**Authors:** Kuntong Jia, Yongyi Peng, Xueji Chen, Huahua Jian, Min Jin, Zhiwei Yi, Ming Su, Xiyang Dong, Meisheng Yi

**Affiliations:** a School of Marine Sciences, Sun Yat-sen University, Guangzhou, Guangdong, China; b Key Laboratory of Marine Genetic Resources, Third Institute of Oceanography, Ministry of Natural Resources, Xiamen, Fujian, China; c Guangdong Provincial Key Laboratory of Marine Resources and Coastal Engineering, Guangzhou, Guangdong, China; d Southern Marine Science and Engineering Guangdong Laboratory (Zhuhai), Zhuhai, Guangdong, China; e State Key Laboratory of Microbial Metabolism, Joint International Research Laboratory of Metabolic and Development Sciences, School of Life Sciences and Biotechnology, Shanghai Jiao Tong University, Shanghai, China; University of Minnesota

**Keywords:** *Alteromonas*, *Inoviridae*, isolation, bacteria-phage interactions, transcriptome

## Abstract

Members from the *Inoviridae* family with striking features are widespread, highly diverse, and ecologically pervasive across multiple hosts and environments. However, a small number of inoviruses have been isolated and studied. Here, a filamentous phage infecting Alteromonas abrolhosensis, designated ϕAFP1, was isolated from the South China Sea and represented a novel genus of *Inoviridae*. ϕAFP1 consisted of a single-stranded DNA genome (5986 bp), encoding eight putative ORFs. Comparative analyses revealed ϕAFP1 could be regarded as genetic mosaics having homologous sequences with *Ralstonia* and *Stenotrophomonas* phages. The temporal transcriptome analysis of A. abrolhosensis to ϕAFP1 infection revealed that 7.78% of the host genes were differentially expressed. The genes involved in translation processes, ribosome pathways, and degradation of multiple amino acid pathways at the plateau period were upregulated, while host material catabolic and bacterial motility-related genes were downregulated, indicating that ϕAFP1 might hijack the energy of the host for the synthesis of phage proteins. ϕAFP1 exerted step-by-step control on host genes through the appropriate level of utilizing host resources. Our study provided novel information for a better understanding of filamentous phage characteristics and phage-host interactions.

**IMPORTANCE** *Alteromonas* is widely distributed and plays a vital role in biogeochemical in marine environments. However, little information about *Alteromonas* phages is available. Here, we isolated and characterized the biological characteristics and genome sequence of a novel inovirus infecting Alteromonas abrolhosensis, designated ϕAFP1, representing a novel viral genus of *Inoviridae*. We then presented a comprehensive view of the ϕAFP1 phage-Alteromonas abrolhosensis interactions, elucidating reprogramed host metabolism and motility. Our study provided novel information for better comprehension of filamentous phage characteristics and phage-host interactions.

## INTRODUCTION

Bacteriophages, the viruses of bacteria, are the most abundant biological entities in the global ecosystem. The complex, dynamic phage-bacteria interactions make phages have dramatic influences on the diversity and physiology of their hosts, the marine nutrient cycle, and horizontal gene transfer (1, 2). Most of the known phages belong to the order *Caudovirales* which are tailed phages with an isometric capsid and divided into three phylogenetically related families: *Myoviridae*, *Siphoviridae*, and *Podoviridae*. Only a few phages accounting for less than 4% of all known phages are shaped as polyhedral, filamentous, or pleomorphic (3). Among them, filamentous bacteriophages belonging to *Inoviridae*, *Lipothrixviridae*, and *Rudiviridae* families, are relatively rare (4, 5).

All phages from the *Inoviridae* family (inoviruses) share a similar filamentous virion shape which is typically about 6 to 8 nm in width and 800 to 2000 nm in length containing a circular single-stranded DNA (ssDNA) genome of nearly 4 to 12 kb (6–8). The genome of filamentous phage encodes around 10 genes, which are composed of four functional modules involved in genome replication, virion structure, assembly/secretion, and regulation (9). Almost all the described filamentous phages were isolated from Gram-negative bacteria (10), while only two filamentous phages were found in the Gram-positive bacteria, Propionibacterium freudenreichii and Clostridium acetobutylicum (11). Unlike the typical bacteriolytic-tailed phages, filamentous phages can produce new mature virions which are even packaged on the cell surface and then secreted to the extracellular space without killing the host bacteria (12). In addition, like many other phages, filamentous phages can undertake wide-ranging DNA recombination to catch new genes and perform as important shuttles for gene transfer among host cells, which may promote bacterial evolution (13, 14).

The genus *Alteromonas* contains species of marine bacteria that are described as Gram-negative, strictly aerobic and globally oceanic-distributed heterotrophic bacteria with an unsheathed single polar flagellum (15). Members of this genus are widely found in surface seawater, open deep ocean, and coastal seawater (16, 17). Despite the *Alteromonas* strains being virtually ubiquitous and playing a crucial role in the biogeochemical cycling of marine ecosystems, the knowledge regarding the phage-host interaction is still poor, especially the impact of temperate phages infecting *Alteromonas*.

Phages take over host resources for reproduction by manipulating hosts’ critical biological pathways involved in transcription, translation, signal transduction, and metabolism (18, 19). However, the relationship between filamentous bacteriophages and their hosts is not only antagonistic but also mutualistic in many aspects. Several researchers have reported some filamentous bacteriophages can affect the virulence factor and pathogenicity of the host by carrying cholera toxin genes or activating hosts’ virulence genes (20, 21). Moreover, filamentous bacteriophages may be beneficial to hosts’ adaptive strategies in the deep-sea environment, which have been shown to significantly regulate the synthesis of transcription and translation elements, as well as the swarming motility of deep-sea bacteria for improved host survival (22, 23). In addition, Pseudomonas aeruginosa phage Pf1 has been known to enhance the resistance of biofilm by their hosts to environmental stress (24).

Relative to the abundant existence of *Alteromonas* strains in the marine environment, the sparsely reported phages infecting *Alteromonas* showed a huge contrast. In this work, we isolated and purified a new filamentous phage, ϕAFP1, that infected the Alteromonas abrolhosensis strain from a seawater sample collected from the South China Sea. We characterized its morphology, biological characteristics, genomic features, and gene structure-function analysis. We further investigated the first global glimpse of the interactions between *Inoviridae* and *Alteromonas* by a combination of whole-genome sequencing and RNA sequencing (RNA-seq). This study expanded our understanding of *Alteromonas* phage diversity in the marine environment, together with the genomic characteristics and phage-bacterium interaction of filamentous phages.

## RESULTS

### Morphology and characterization of ϕAFP1.

**FIG 1:**
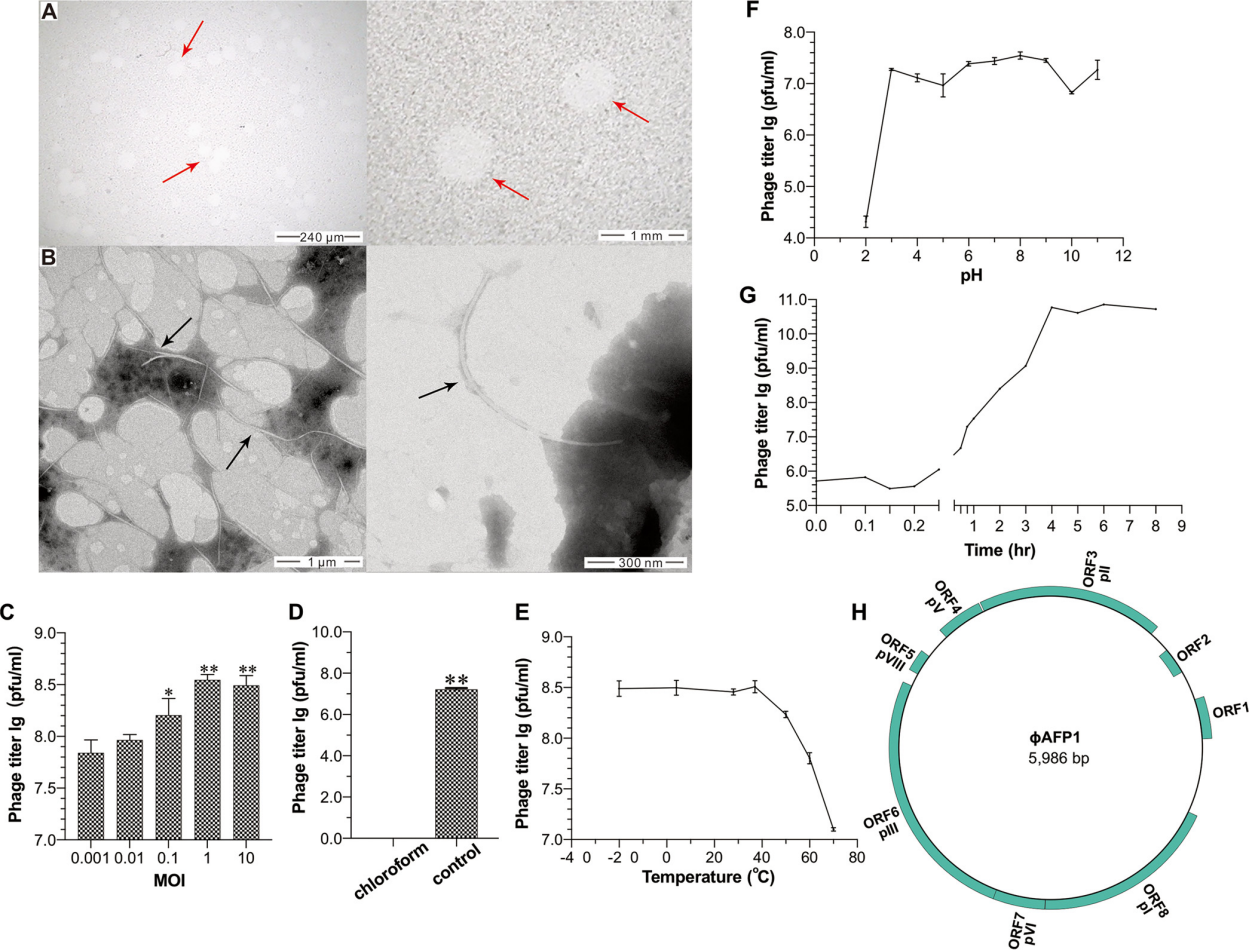
Plaque, morphology, and biological characterization of phage ϕAFP1. (A) The observations of plaques formed on the lawn under different magnifications. Red arrows indicated the plaques of phage ϕAFP1 against the lawn of the host bacterium A. abrolhosensis. (B) Transmission electron microscopy observations of phage ϕAFP1 negatively stained. The black arrows indicated the phages. (C) Optimal multiplicity of infection (MOI) value of phage ϕAFP1. (D) Sensitivity of phage ϕAFP1 to chloroform. (E) Biological stability of phage ϕAFP1 under different temperature conditions. (F) Sensitivity of phage ϕAFP1 to pH from 2 to 10. (G) One-step growth curve of phage ϕAFP1. The mean titer is shown from triplicate assays. The asterisk indicates significant differences between groups (*, *P* < 0.05; **, *P* < 0.01) using One-way ANOVA. (H) Circular diagram of phage ϕAFP1 genome and predicted ORFs. Rects indicate ORFs in the plus or minus strand.

A filamentous phage infecting A. abrolhosensis strain JKT1, named ϕAFP1 was isolated from the South China Sea. Phage ϕAFP1 formed turbid, circular, and boundary smooth plaques with 0.5 to 1.0 mm in diameter in a double agar layer at 28°C while no plaque appeared in control (Fig. 1A). The morphology of ϕAFP1 was observed by transmission electron microscopy (TEM) and appeared to be filamentous in shape approximately 10 nm in width and 900 nm in length (Fig. 1B).

The multiplicity of infection (MOI) analysis showed that infection with phages at an MOI of 1 produced the highest phage titer (3.51 × 10^8^ PFU mL^−1^) (Fig. 1C). The infectivity of phage ϕAFP1 was completely inactivated after chloroform treatment (Fig. 1D). Phage ϕAFP1 was stable and retained nearly 100% infectious activity in a range from −20°C to 37°C but had a continuous and rapid decline in surviving phages titer when the temperature was higher than 37°C (Fig. 1E). ϕAFP1 grew well at pH values from 3 to 9 but had a substantial decline in phage titer with sharply decreased to 0.11% at pH 2 and 23.99% at pH 10, respectively (Fig. 1F). The latent period of ϕAFP1 was approximately 12 min and then there was an ascent stage which continued for 228 min before entering the plateau period (Fig. 1G). The burst size of ϕAFP1 presented that about 10 phage particles were produced per infected host cell. The agarose gel electrophoresis revealed that the genome size of ϕAFP1 was around 5 to 6 kb (Fig. S1A in Supplemental File 1). The genome was resistant to general restriction enzymes and RNase A but sensitive to DNase I and S1 nuclease (Fig. S1B and C in Supplemental File 1), suggesting ϕAFP1 consists of ssDNA. The purified ϕAFP1 particles had a major protein band with theoretical molecular masses of ~4 kDa visible on a Tris-tricine-SDS-PAGE (Fig. S1D in Supplemental File 1).

To examine the host range of phage ϕAFP1, the potential hosts were predicted using sequence-based tools PHIS Detector, and there was not any predicted host found.

### Genomic analysis and annotation of ϕAFP1.

The genome of phage ϕAFP1 composed of 5986 bp with 40.34% G+C content, the completeness was estimated as 100% in CheckV. ϕAFP1 was predicted to encode eight open reading frames (ORFs) accounting for 82.69% of the genome, including three capsid proteins, two replication-related proteins, a zonular occludens toxin (ZOT)-like protein, and two proteins of unknown function (Fig. 1H and Table S1). ϕAFP1 contained a small amount of ORFs compared to other inoviruses (Table S2 in Supplemental File 2).

**FIG 2:**
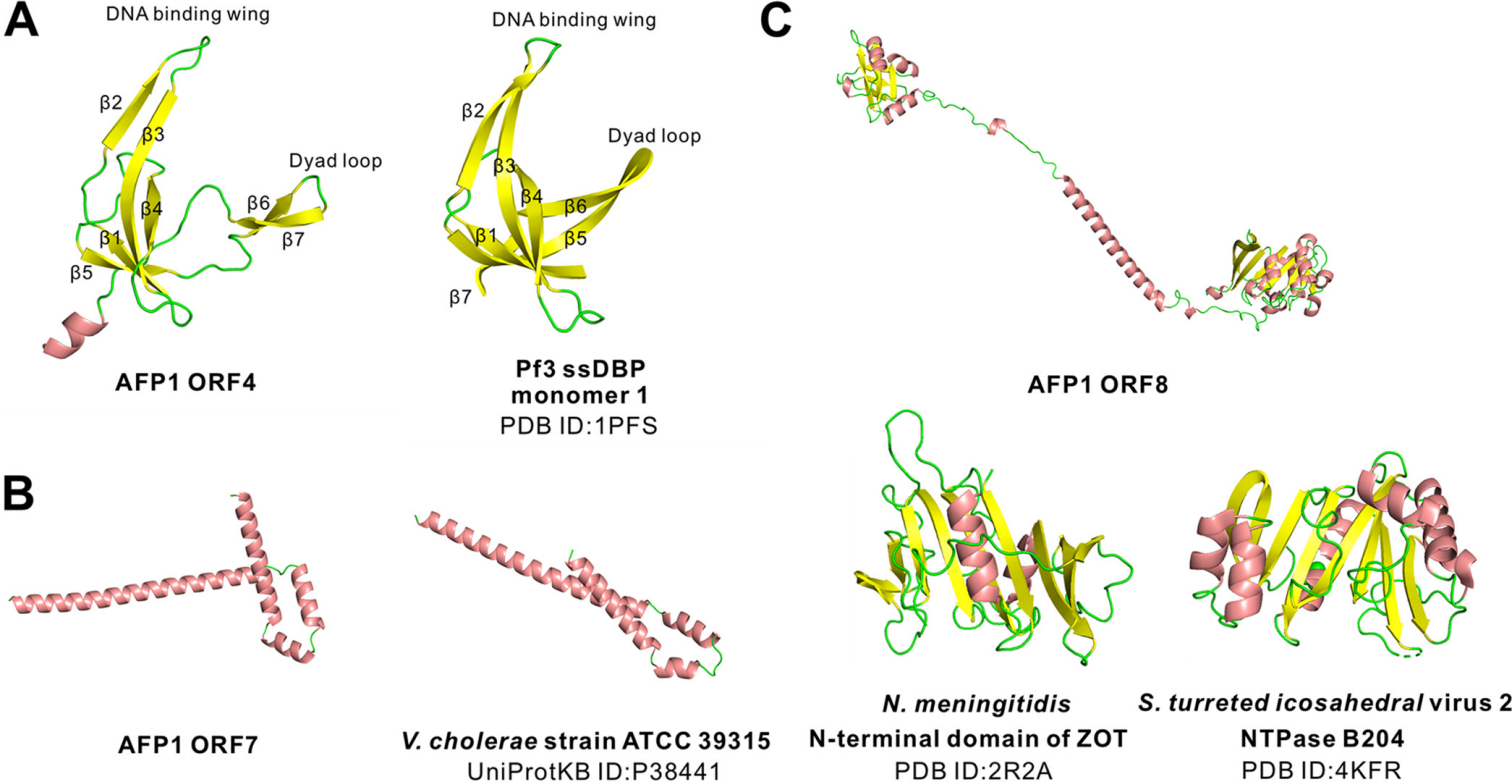
Predicted structures with the highest confidence of ORFs proteins from ϕAFP1 and their homologous proteins. (A) ORF4 from ϕAFP1 and the ϕPf3 homolog (PDB accession no. 1PFS, monomer 1). (B) ORF7 from ϕAFP1 and the V. cholerae strain ATCC 39315 homolog (UniProtKB accession no. P38441). (C) ORF8 from ϕAFP1 and its homologs from the N. meningitidis N-terminal domain of ZOT (PDB accession no. 2R2A) and S. turreted icosahedral virus 2 NTPase B204 (PDB accession no. 4KFR).

Three-dimensional structures of ϕAFP1 ORFs were generated to predict the protein function using RoseTTAFold. As shown in Fig. 2A, the predicted ORF4 consisted of seven β-strands, the four-stranded antiparallel sheet (β1, β3, β4, and β5) across the molecule, and two prominent hairpins were designated the DNA binding wing and dyad loop, respectively. Its structure was similar to the monomer of a homodimer single-stranded DNA binding protein (ssDBPs, pV) (PDB accession no. 1PFS) encoded by the filamentous phages ϕPf3. The secondary structure of ORF3 (putative phage/plasmid replication protein, pII) defined two domains, including obvious sandwich domain (OD) (amino acids 1 to 265) and terminal domain (TD) (amino acids 276 to 346) (Fig. S2C in Supplemental File 1). OD folds as an α-β sandwich with central eight-antiparallel β-strands inserted with six helices flanking on two sides. TD was a three-helix bundle with one of the helices nearly perpendicular to the others. The ORF7 contained a four-helix bundle with one of these helices nearly perpendicular to others (confidence, 0.79) and partly resembles the protein of unknown function DUF2523 (Fig. 2B), which was also identified as accessory cholera enterotoxin (P38441) in Vibrio cholerae, suggesting the putative role of ORF7 involving in the assembly of the ϕAFP1 virion at the membrane. The ORF8 in ϕAFP1 encoded a predicted ZOT. The N terminus of ORF8 adopted an αβα-fold, where eight-parallel β-strands (one was antiparallel) with six α-helices on one face and six α-helices on the opposite face, had fewer similarities in the N terminus of Zot from Neisseria meningitidis. Furthermore, the considerable similarity of structural prediction of ORF8 to the P-loop containing nucleoside triphosphate hydrolase superfamily was observed (Fig. 2C).

### ϕAFP1 represented a novel viral genus of *Inoviridae* and was a genetic mosaic.

**FIG 3:**
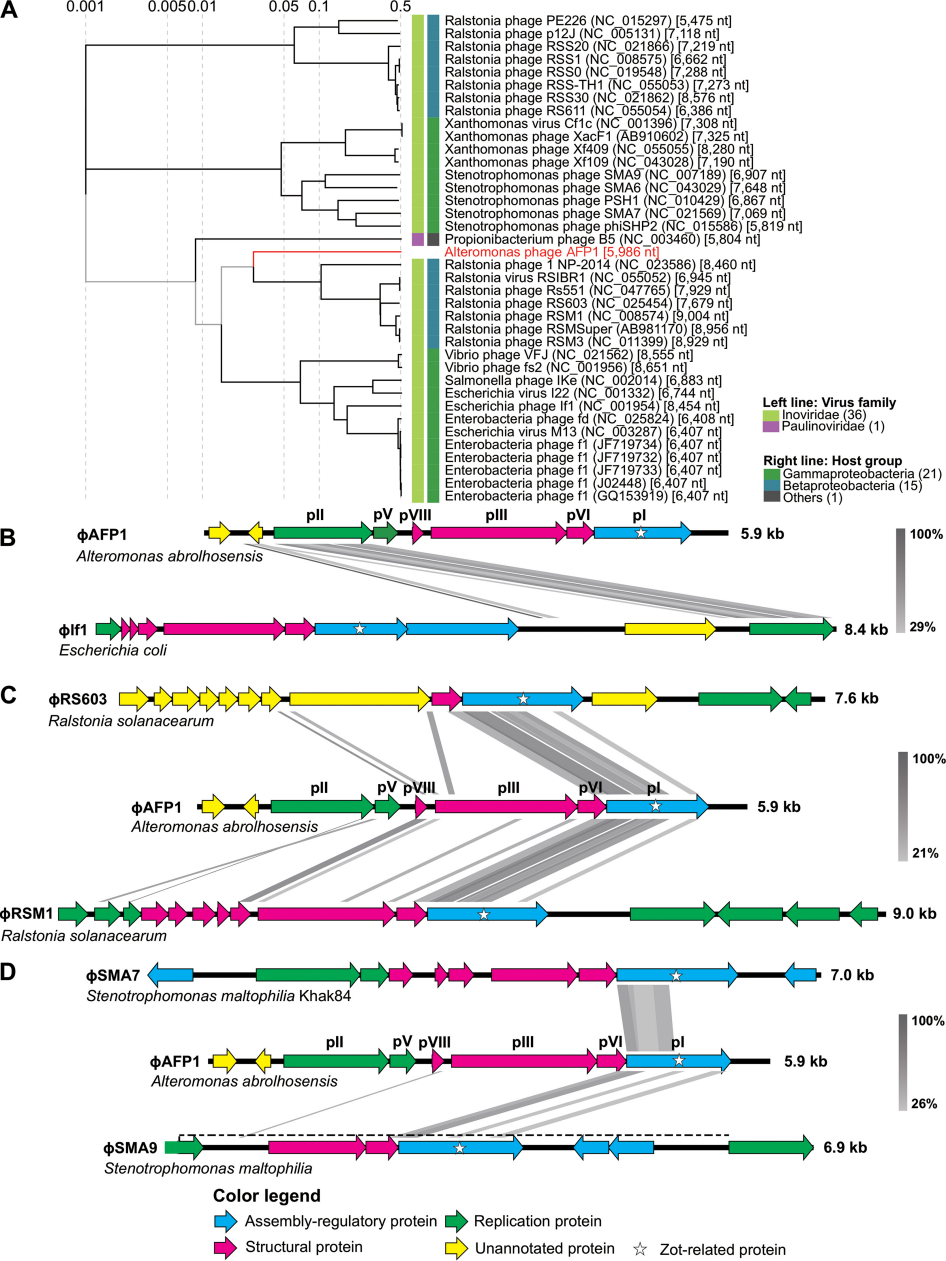
Phylogenetic trees and comparative analysis of ϕAFP1. (A) Phylogenetic tree generated based on amino acid sequences of complete genomic sequences of ϕAFP1 and other different filamentous phages in *Inoviridae* and *Paulinoviridae* family. Further details for phages represented on the tree were listed in Table S3 in Supplemental File 2. (B to D) Comparative analysis of the linear genomic organization of ϕAFP1 and other three kinds of filamentous phages. Arrows with different colors represent the putative ORFs belonging to functional modules (replication module, green; Structure module, red; Assembly regulatory module, blue; unannotated genes, yellow). Further details for comparative analysis were listed in Table S3 in Supplemental File 2.

To investigate the evolutionary history of ϕAFP1, a phylogenetic tree was constructed based on the amino acid sequences of the whole complete genome from 36 *Inoviridae* and one *Paulinoviridae* (Table S4 in Supplemental File 2). As shown in Fig. 3A, ϕAFP1 was closely related to *Ralstonia* phage but far from other characterized *Inoviridae* viruses. Network analysis of the ϕAFP1 genome together with the publicly available virus genomes showed that the ϕAFP1 sequence was disconnected from other viral clusters at the genus level (Table S5 in Supplemental File 2). These results suggested that ϕAFP1 presented a new genus of *Inoviridae*.

To investigate the origin of ϕAFP1 and identify the function of its ORFs, the homology of sequences with other *Inoviridae* viruses was performed by blastp (Table S2 in Supplemental File 2). The genomic organization of phage ϕAFP1 was modular, including a replication module, structural module, and assembly regulatory module. ORF3 showed sequences similar to the If1p10 of *Enterobacteria* phage If1 with the top hit (52.4% amino acid similarity) (Fig. 3B), which was predicted to represent phage and plasmid replication proteins (gene II/X family). ORF4 had a certain level of similarity to ORF2 (ssDBP) of *Stenotrophomonas* phage, phiSMA6 (Fig. S3A in Supplemental File 1). ORF5 had the highest sequences similarity with major capsid protein (pVIII) of *Ralstonia* phages (48.83%) (Fig. 3C and Fig. S3B in Supplemental File 1). ORF7 was aligned to the minor coat protein pVI of *Ralstonia* phages (Fig. 3C and Fig. S3B in Supplemental File 1). The amino acid sequence of ORF6 had 27.10%, 27.06%, and 28.70% identity to the ORFs from *Ralstonia* phage RSM1, RSMSuper, and RS603 (Fig. 3C and Fig. S3B in Supplemental File 1), respectively. Within the assembly regulatory module, ORF8 of ϕAFP1 was predicted to encode the pI-like gene (the most conserved gene in inoviruses), the certain level of identity of ORF8 was found both in *Ralstonia* and *Stenotrophomonas* phages (Fig. 3 and Fig. S3 in Supplemental File 1). The additional ORF1 and ORF2 had no significant similarity detectable to sequences in the databases. These results suggested that ϕAFP1 was a genetic mosaic. Furthermore, the close relationships were verified by a phylogenetic tree based on the amino acid sequences of Zot-like proteins, showing that ϕAFP1 was more related to *Ralstonia* phage and *Stenotrophomonas* phage (Fig. S4 in Supplemental File 1).

### A temporally coordinated program of ϕAFP1 gene expression.

To establish characteristic responses of the host infected by ϕAFP1, we first assembled the high-quality genome of A. abrolhosensis strain JKT1 (4,402,100 bp, GC 44.67%) from short and long sequencing reads with estimated completeness of 100% and contamination of 0.3%, and 3573/3699 genes were annotated in the bacterial genome (Table S6 in Supplemental File 2).

**FIG 4:**
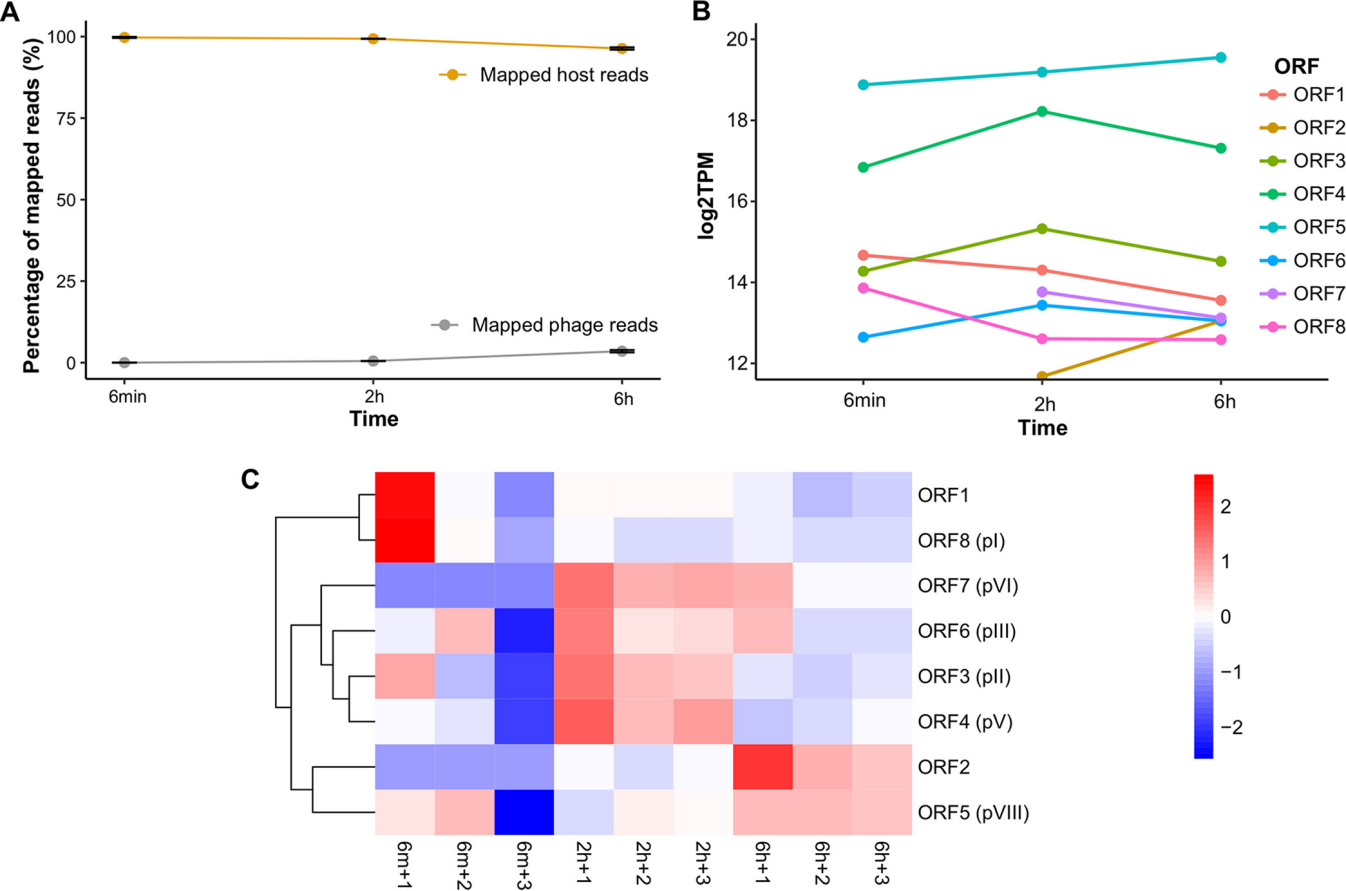
Temporal transcriptional profile of ϕAFP1. (A) Alignment of RNA read sets against the A. abrolhosensis (orange) or ϕAFP1 (gray) genome at three time points after infection. Data are displayed as the means SD from three independent experiments. (B) The relative abundance of the expression of phage ORFs at three time points. (C) Hierarchical cluster heat map of the expression level of ϕAFP1 ORFs based on the TPM values of each gene. Further details for reading alignment and expression abundance of phage ORFs can be found in Table S7 and S8 in Supplemental File 2.

Based on the one-step growth curve of ϕAFP1, approximately three-time points (6 min, 2 h, and 6 h) for latent, burst, and plateau periods that spanned the entire infection cycle duration were selected as representative snapshots for exploring the gene expression patterns of both ϕAFP1 and host A. abrolhosensis by RNA-seq. The transcriptome of host A. abrolhosensis strain JKT1 during the phage infection cycle was progressively taken the place of phage ϕAFP1 transcripts, fluctuating from 0.0% at 6 min and peaking at 3.5% at 6 h (Table S7 in Supplemental File 2), and the proportion of host reads reached a very high percentage around 96.3% to 99.7% (Fig. 4A).

According to the expression profiles of the individual genes (Fig. 4B, Table S8 in Supplemental File 2), ORF4 kept a relatively high expression level among all ϕAFP1 genes and represented the active replication of viruses during phage infection. The temporally coordinated program of phage gene expression could be clustered into early, middle, and late categories. ORF1 and ORF8 were highly expressed at 6 min and decreased thereafter, the abundance of ORF3, ORF4, ORF6, and ORF7 was at its peak at 2 h, whereas genes expressed late were the ORF2 and ORF5 (Fig. 4B and 4C).

### ϕAFP1 reprogramed host metabolism and motility.

**FIG 5:**
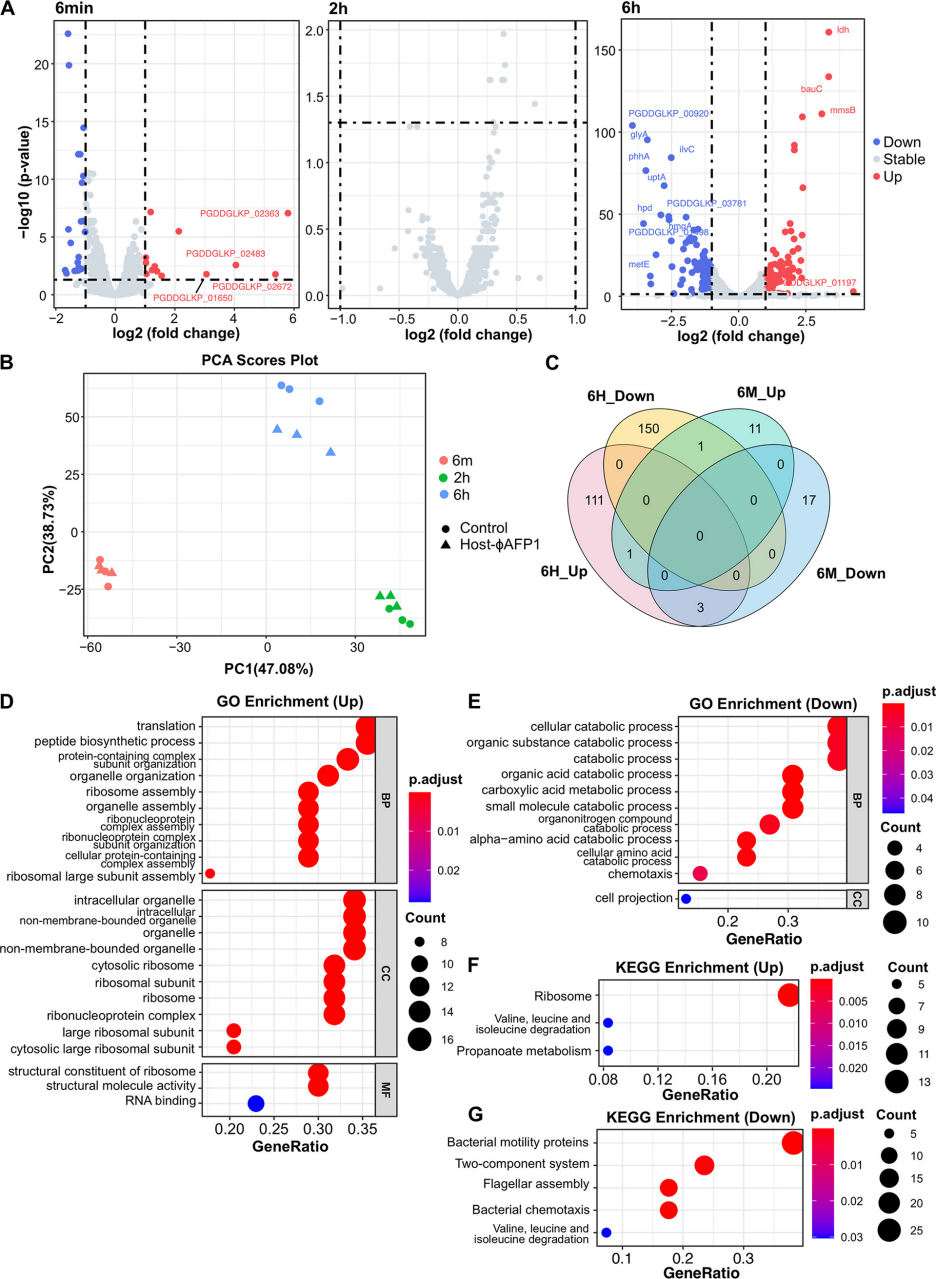
Impact of ϕAFP1 infection on its host transcriptome. (A) Volcano plot of the A. abrolhosensis transcriptome following phage infection compared with the uninfected control at three infection stages. (B) PCA plot of the expression of A. abrolhosensis genes in phage-infected or phage-uninfected groups. (C) Number and intersection of upregulated or downregulated DEGs at two infection stages. Significant enrichment GO analysis (D and E) and KEGG (F and G) categories of host DEGs (upregulated and downregulated genes) enriched at 6 h after ϕAFP1 infection (adjusted *P* < 0.05). The enrichment-adjusted *P* value of each pathway is shown as a color gradient. The number of genes enriched in each pathway is represented by the size of the points. Down, downregulated DEGs; Up, upregulated DEGs; Stable, nonsignificantly changed genes. Further details for DEGs of the host can be found in Table S9, S12, and S13 in Supplemental File 2.

To investigate the responses of host A. abrolhosensis to phage ϕAFP1 infection, we analyzed the differentially expressed genes (DEGs) and pathways in the host throughout the infection cycle. Totals of 0.87% (33/3779), 0% (0/3779), and 7.04% (266/3779) genes were differentially expressed at 6 min, 2 h, and 6 h in response to phage infection, respectively (Fig. 5A and Table S9 in Supplemental File 2). The differential fold change of DEGs measured by RT-qPCR was in good agreement with the RNA-seq data, including upregulated DEGs (*mmsB*, *prpB,* and *phoB*) and downregulated DEGs (*fadA*, *flgE*, *flgL*, *flgG*, *hppD*, *prpC*, and *galK*) (Fig. S5 in Supplemental File 1 and Table S10 in Supplemental File 2).

The expression of A. abrolhosensis genes changed greatly during ϕAFP1 infection, according to PCA (principal component analysis) based on the TPM (transcript per million) values of host genes (Fig. 5B). The most highly expressed genes at all the time points during infection encoded DNA binding and ribosomal-related proteins, which was varied among three groups (Fig. S6 and S7 in Supplemental File 1, Table S11 in Supplemental File 2). Most of the DEGs were specific at 6 min and 6 h (except for upregulated DEG *cyoB*), and only four DEGs undulated between upregulation and downregulation (*hutD*, *prpB*, *yegQ* and gene encoding COG0517 signal transduction mechanisms domain) (Fig. 5C). Moreover, the great majority of the DEGs were downregulated throughout infection, indicating the negative control by ϕAFP1 on host genes.

Next, 10 DEGs (10/33) at 6 min were annotated in gene ontology (GO) and enriched in KEGG (Kyoto Encyclopedia of Genes and Genomes) pathways. Annotated upregulated genes were *gntX* and *cyoB*, while part of downregulated genes at 6 min encoded transport-related proteins (e.g., ExbD/TolR and BtuB), organic acid-related metabolic proteins (e.g., PrpB, GalK, PrpC, and HutU) (Table S9). However, our result showed that none of the DEGs were found at 2 h post ϕAFP1 phage infection (the burst period) compared to the uninfected host (Table S9 in Supplemental File 2).

To gain the biological functions of the significantly changing host genes, 83/266 DEGs at 6 h were annotated with GO function by Eggnog-mapper, showing the remarkable function classifications of annotated DEGs (Fig. 5D and E, Table S12 in Supplemental File 2). Most upregulated genes of the 6 h group were enriched in translation, ribosomal structure, ribosome and organelle biogenesis, and transcription, revealing that ϕAFP1 highly demanded the translation machinery of the bacterial host. Most of the downregulated genes were involved in the pathways of chemotaxis and organic substance catabolic process (3.4-fold change gene *phhA*; 2.6-fold change gene *hmgA*), including organic acid, small molecule, organonitrogen, and amino acid.

Of the DEGs at 6 h, 62.4% (166/266) clustered as 68 KEGG pathways, and the pathway categories of upregulated and downregulated DEGs were marked, respectively (Fig. 5F and G, Table S13 in Supplemental File 2). The upregulated DEGs were enriched in ribosome, valine, leucine, and isoleucine degradation and propanoate metabolism pathways. The downregulated DEGs were mainly enriched in bacterial motility proteins, a two-component system, flagellar assembly, bacterial chemotaxis, valine, leucine, and isoleucine degradation pathway. Several DEGs involved in valine, leucine, and isoleucine degradation pathway also participated in different metabolic processes, such as the downregulated formation (*ilvE*) and upregulated degradation (*pdhA*) of methyl-2-oxobutanoate (Fig. S8 in Supplemental File 1). A total of 27 downregulated DEGs in A. abrolhosensis strain JKT1 were related to bacterial motility pathways, including genes related to flagellar assembly (e.g., *fla*, *flg*, and *fli* family) (Fig. S9 in Supplemental File 1), pilus assembly protein (PilY1 and PilX), and chemotaxis genes (*cheA*, *cheW*, *cheY*, and *aer2*) (Fig. S10 in Supplemental File 1), suggesting the reduction of flagella dependent cell movement caused by ϕAFP1.

## DISCUSSION

In this study, we reported the isolation and complete genome sequence of a filamentous phage designated ϕAFP1 from the South China Sea using the A. abrolhosensis strain JKT1 as the host bacterium. Until now, only 10 published reports about bacteriophages isolated from *Alteromonas* were available, including *Siphoviridae* phages PB15, JH01, P24, XX1924, and R7M and R9Y-phages (25–29), *Myoviridae* phage V22 (30), *Podoviridiae* phages altaD45 P1 to P4 (31) and *Mareflavirus* phage ZP6 (32), *Autographiviridae* phages H4/4’ (33) and R8W (34). Different from the above phages, ϕAFP1 consisted of a flexible filament (width = 10 nm, length = 900 nm), suggesting that this phage was a member of the family *Inoviridae*. To date, only two filamentous phages infecting bacteria of the family *Alteromonadaceae* have been isolated and reported, including phage f327 infecting *Pseudoalteromonas* (22) and SW1 infecting *Shewanella* (35). To the best of our knowledge, this was the first report of the filamentous phage infecting *Alteromonas*. The family *Inoviridae* includes 21 genera and 27 species with genomes of about 5.5 to 10.6 kb encoding 7 to 15 proteins (8). ϕAFP1 contained fewer coding genes (8 ORFs) and had a small genome size (5.9 kb) with the smallest burst sizes (10 PFU/cell) compared with other currently known *Inoviridae* and alterphages (Table S3 in Supplemental File 2). The genus demarcation of inoviruses was set at 50% amino acid identity on the pI-like gene (i.e., Zot) and the major capsid protein, and there was no significant similarity of both Zot and major capsid proteins found between ϕAFP1 and other inoviruses. In addition, the phylogenetic analysis and gene-sharing network suggested that ϕAFP1 represents a new viral genus of *Inoviridae.*

One of the most evident features of bacteriophage genomes is their genetic mosaicism (36). Previous studies have observed mosaicism within several phages, such as Pseudomonas phages (37–39), *Actinobacteria* phages (40), and *Mycobacteria* phages (41). Phage mosaic genomes are relatively common in temperate phages, revealing the horizontal exchange of genetic material between distinct phages and phages or their hosts. Inoviruses genomes are often shaped by frequent recombination and a mosaic of genes emerged as a result (42–44), which plays a key role in the evolution of these viruses by leading to the acquisition of nonorthologous genes or gene exchange among highly divergent orthologs (45, 46). Here, the comparative genomic analysis demonstrated the mosaic nature of the ϕAFP1 genome, which was closely related to *Ralstonia* phages and *Stenotrophomonas* phages. The gene encoding Zot-like protein in ϕAFP1 was similar to that in both these phages, while the structure-related genes were more homologous with genes from *Ralstonia* phages. The genetic exchange may yield new combinations of genes and protein domains or give rise to nonfunctional genomic trash (47). These exchange events among ϕAFP1, *Ralstonia* phages, and *Stenotrophomonas* phages result in the inclusion of key genes of inovirus genomic modules, we speculate that there might be frequent communication between *Ralstonia* phages and *Stenotrophomonas* phages. Although we did not identify novel hosts of ϕAFP1 in this study, considering that genetic recombination more likely occurs between phages whose host ranges are more similar, we predicted that the hosts of *Ralstonia* phages and *Stenotrophomonas* phages might also be the potential hosts of ϕAFP1.

The lysogenic phage pattern of ϕAFP1 exerted step-by-step control on host genes, especially on genes belonging to DNA, RNA, and protein biosynthesis, central metabolism, and bacterial motility. The upregulation of translation processes related to DEGs at 6 h indicated ϕAFP1 hijacked cellular resources for the assembly of phages. The findings that phages hijacked the synthesis systems of the hosts were also observed in other phages (19, 48, 49). In contrast, it was reported that phage infection could stop the host macromolecular synthesis promptly and completely (50). Pioneering work on bacterial motility revealed that phages could exert multiple roles in their hosts, including contributing to searching more adaptive environments, serving as virulence factors for pathogens, and being conducive to symbiotic relationships (51–54). The downregulation of host genes related to material catabolic processes and bacterial motility-related pathways was consistent with other temperate phage infections (18, 23, 55, 56). While filamentous phage f327 was reported to improve the motility of the host in Arctic Sea ice (22). Considering that there is a large amount of energy cost for flagellar biosynthesis, bacterial motility, and chemotaxis for the hosts, thus downregulating the key genes of these processes may be useful for ϕAFP1 utilizing cellular resources.

There is a range of responses in different phage-bacterium systems, and the trend of expression changes depends on the host-phage interactions (57). Our global analysis of host regulation indicated that the infection of ϕAFP1 did not interfere with host regulation at the burst period (Fig. 5), which may be the exception rather than the rule known so well for phage infection, that is, most phages will affect hosts’ gene expression to a great extent at its burst period (18, 57, 58). It was observed that ϕAFP1 exerted a temperate infection on A. abrolhosensis, performed with a long release period, relatively low burst size (10 PFU cell^−1^), quite minor proportions of phage transcripts and some upregulated host material metabolism processes to promote the use of biomolecules. Therefore, it is supposed that the distinctive shifts in the expression of host genes during the infection may be caused by the small and simple genome of ϕAFP1, as well as its unique chronic infection. However, the precise mechanism of ϕAFP1 infection needs to be further studied.

In conclusion, the novel *Inoviridae* phage ϕAFP1 isolated from the South China Sea was the first report of the filamentous phage infecting *Alteromonas*. Its biological and genomic characterization information could provide new information for the virology of *Inoviridae*. Furthermore, we found diverse and novel expression patterns of phage and host at different time points during infection, which reveal reprogramming of the host metabolism and repression of the bacterial motility system. Our results will contribute to a broader understanding of phage-bacterium interaction.

## MATERIALS AND METHODS

### Bacterial strains and culture conditions.

The host A. abrolhosensis strain JKT1 was isolated from seawater (20 cm deep) collected from Tangjia Bay (Zhuhai, Guangdong, China) and was grown at 28°C on 2216E marine agar medium (Hopebio, China).

### Phage isolation, purification, and concentration.

The seawater sample used for ϕAFP1 isolation was collected from the northern sea area of Xisha Islands (Paracel Islands) in the South China Sea (17°04′37.27″N, 111°28′24.87″E) during the summer of 2019. The phage isolation assay was performed as described previously with some modifications (59). The seawater sample (5 mL) was added to an equal volume of 2216E liquid medium and inoculated with 750 μL of A. abrolhosensis strain JKT1 overnight culture in an incubator shaker (28°C) (Crystal, USA) for 18 h. Then, the culture was centrifuged, and the supernatant was filtered through a 0.22 μm-pore-size sterile PES syringe filter (Sorfa, China) to remove bacterial cells and debris.

Phage was detected by the soft agar overlay method with some modifications (60). The filtered solution was incubated with A. abrolhosensis strain JKT1 at RT for adsorption, mixed with soft agar, and then poured on a bottom agar plate immediately. After overnight cultivation at 28°C, the obvious plaques were picked and suspended in SM buffer. Six rounds of plaque isolation experiments were carried out to acquire purified phages, then which were stored in SM buffer at 4°C.

Phage ϕAFP1 was condensed by the standard protocol described by Sambrook and Russel (61). Overnight culture of phage ϕAFP1 and A. abrolhosensis *strain* JKT1 (1 L) was pelleted at 15,000 × *g* for 10 min. The supernatant was mixed with polyethylene glycol 8000 and bathed on the ice for 2 h. Phage particles were precipitated at 10,000 × *g* for 10 min at 4°C, after which the pellet was resuspended by SM buffer followed by the addition of cesium chloride (0.75 g mL^−1^) (CsCl, Sigma-Aldrich, USA). The mixture was transferred to an ultraclear centrifuge tube (Beckman Coulter) and centrifuged in an Optima XPN-100 Ultracentrifuge (Beckman Coulter, USA). Finally, the phage pellet was resuspended with SM buffer and kept at −80°C for use.

### Morphological observation.

One drop of high-titer purified phage ϕAFP1 solution was subjected to carbon-coated copper grids (400 mesh) for adsorption for 2 min before negatively stained with 2% (wt/vol) phosphotungstate (pH 7.0), then observed with a JEM-1400 electron microscope (JEOL Ltd., Japan) at a working voltage of 120 kV.

### Host range preducation.

The sequence-based tools PHIS Detector (http://www.microbiome-bigdata.com/PHISDetector/) were used for detecting phage-host interactions (62).

### Biological characteristics and bacteriolytic activity *in vitro*.

Thermal and pH stability and chloroform sensitivity were tested as described previously (25). For thermal stability, phage suspension (10^8^ PFU mL^−1^) was sustained separately at different temperatures (–20°C, 4°C, 28°C, 37°C, 50°C, 60°C, and 70°C) for 2 h. For pH stability, phage stock (10^8^ PFU mL^−1^) was individually added into SM buffer with different pH values from 2 to 10 and then incubated for 2 h. To assess the influence of chloroform on ϕAFP1, chloroform was added into an aseptic tube containing phage stock (10^8^ PFU mL^−1^).

The MOI assay was performed as described previously with some modifications (63). The phage stock solution was diluted in a gradient with SM buffer and then infected A. abrolhosensis strain JKT1 (10^8^ CFU mL^−1^) at MOIs of 0.001, 0.01, 0.1, 1, or 10 for 20 min at RT, respectively.

### One-step growth curve.

A one-step growth curve assay was performed as described previously with some adjustments (64). A. abrolhosensis strain JKT1 culture (10 mL, optical density at 600 nm [OD_600_] = 0.4 to 0.5) was harvested by centrifugation (2,500 × *g*) at RT for 10 min. Then, the pellet was resuspended in SM buffer and added with phage stock solution (10^9^ PFU mL^−1^) at an MOI of 1. After 20 min standing at RT, the mixture was centrifuged (2,500 × *g*) at RT for 5 min and washed with SM buffer three times to remove free phage particles. The pelleted mixture was carefully resuspended in 2216E liquid medium, then incubated in a shaker (180 rpm) for 8 h at 28°C. During the cultivation, a phage sample was taken at 3-min intervals for the first 15 min, 15-min intervals for the next 45 min, and 1-h intervals for the remaining 7 h, until 8 h. Every sample was immediately centrifuged (8,000 × *g*) once it was taken, then the supernatant was transferred to a new sterile tube and stored at 4°C temporarily. Finally, the phage titer was determined by the soft agar overlay method mentioned above.

### Genomic DNA and protein analysis.

The genomic DNA of phage ϕAFP1 was extracted using a Viral DNA kit and separated on 1% (wt/vol) agarose gels. For analyzing phage genomic DNA characteristics, phage DNA was treated with DNase I, RNase A, S1 nuclease, and general restriction enzymes.

Tris-tricine-SDS-PAGE was carried out to analyze structural proteins of phage ϕAFP1 (65). The purified phage particles (10^12^ PFU mL^−1^) were mixed with 2 × lysis loading buffer (Solarbio, China) and boiled for 10 min. Thereafter, the prepared sample was separated by electrophoresis on a 20% Tricine-SDS polyacrylamide gel (Solarbio, China) which was then stained with Coomassie brilliant blue R-250 (Amresco, USA) to visualize.

### Sequencing and assembly of phage ϕAFP1.

Whole-genome sequencing of ϕAFP1 was performed using a Truseq SBS kit (300 cycles, Illumina) on Illumina NovaSeq 6000 (Illumina Inc.) at LC Bio (Zhejiang, China). The original sequencing data of phage ϕAFP1 were processed and trimmed by fastp v.0.12.4 to obtain high-quality clean reads (66). Potential viral contigs were assembled by MetaViralSPAdes v.3.15.2 pipeline with default parameters (67). CheckV was used to evaluate the completeness and contamination of assembled genomes of phage ϕAFP1 (68).

### Sequencing and assembly of A. abrolhosensis strain JKT1.

The genomic DNA of A. abrolhosensis strain JKT1 was extracted by Bacterial DNA kit according to the manufacturer’s instructions. The genomes of A. abrolhosensis were sequenced using the PacBio RS II and Illumina NovaSeq platforms. Raw Illumina sequencing reads were trimmed, filtered and quality screened using FastQC v.0.11.8. The assembly of the whole-genome sequence was conducted by Unicycler v.0.4.8, a hybrid assembly pipeline for bacterial genomes (69). The completeness of assembled bacterial genomes was estimated using CheckM (70). The corresponding taxonomy of the bacterial host was assigned by GTDB-TK v202 with reference to GTDB R06-RS202 and classified as A. abrolhosensis strain JKT1 (71).

### Genome annotation.

GeneMarkS-2 (http://opal.biology.gatech.edu/GeneMark/index.html) was used to predict the encoding genes of the assembled genomic sequence of phage ϕAFP1 (72). Functional annotation of A. abrolhosensis strain JKT1 was performed using EggNOG-mapper v.2.1.7 (73) based on eggNOG orthology data (74) and sequence searches were performed using DIAMOND (75).

### Phylogenetic analysis and network analysis.

Whole-genome sequences-based phylogenetic analysis was performed and visualized among all 38 related filamentous phages in GenBank (Table S4 in Supplemental File 2) using ViPTree (76). The sequence similarities of the selected genomes were calculated by ViPTree according to tblastx.

The network analysis of viral genomes was implemented in vConTACT v.2.0 with default parameters to identify genus-level groupings of phage ϕAFP1 (77). The clustered viruses were able to be assigned to the related viral genus and family level in the Viral RefSeq v201 reference database.

### Comparative genomic analysis and structural modeling.

Assembled viral genomes were compared with related reference genomes based on the results from ViPTree and visualized using EasyFig v2.2.2 with the tBLASTx option and the filtering of small hits/annotations option (E value cutoff of 1 × 10^−3^ and min. length of 15 residues) (78). The amino acid identity of proteins between ϕAFP1 and other phages was calculated using BLASTp. Structural modeling for all was performed using RoseTTAFold (79). Protein structures were rendered by PyMoL v.2.5.2 (80).

### Transcriptomics.

Samples (three biological replicates) for transcriptomics were taken in the 3 time points (6 min, 2 h, and 6 h) for latent, burst, and plateau periods of its one-step growth curve. The cultivation of A. abrolhosensis strain JKT1 and ϕAFP1 was performed as described above. Then, the supernatant was decanted, and precipitation was collected. RNA was extracted and quantitated with a Qubit 2.0 fluorometer. The rRNA depletion with the Epicentre Ribo-Zero rRNA Removal kit, library construction with the NEBNext Ultra II Directional RNA Library Prep kit for Illumina, and paired-end sequencing on an Illumina NovaSeq 6000.

RNA-seq reads were quality filtered and trimmed by fastp v.0.12.4, then the ribosomal RNAs in clean data were removed by comparing with rRNA sequences in Rfam and Silva database by SortMeRNA v.4.2.0. The trimmed reads for each library were aligned to the transcripts of A. abrolhosensis strain JKT1 and ϕAFP1 using Bowtie2 v.2.3.5. Preprocessed reads were mapped to the A. abrolhosensis strain JKT1 genome to generate read count quantification TPM (transcripts per million) of each transcript using Salmon v.1.8.0. DESeq2 v.1.30.1 was used to calculate individual transcript expression multiple and pairwise *q* values (false discovery rate rectification by Benjamini and Hochberg method) (81). Fold change was calculated in Excel as expression level in case/expression level in control. Differentially expressed genes were defined as those adjusted *P* ≤ 0.05 and |log_2_(fold change)| ≥1. GO annotation and KEGG enrichment analysis were performed using clusterProfiler v.3.18.1 (82).

### Quantitative reverse transcription-PCR.

Quantitative reverse transcription-PCR (qRT-PCR) was performed to validate the RNA-seq data. The cDNA synthesis was performed with the GoScript Reverse Transcription Mix kit according to the manufacturer’s recommendations. Primers for RT-qPCR are listed in Table S14 in Supplemental File 2. 16S rRNA gene was used as the reference gene for normalization. RT-PCR was performed using Light Cycler 480II real-time PCR system with ChamQ Universal SYBR qPCR Master Mix. The following cycling conditions were used: 45 cycles consisting of 5 min at 95°C, 10 s at 95°C, 35 s at 60°C and 2 min at 40°C.

### Statistics analysis.

All statistical analyses were performed using SPSS (version 19). One-way analysis of variance (ANOVA) was applied for the assessment of the differences between control and treatment groups. The statistically significant and extremely significant differences were represented by *P* < 0.05 (*) and *P* < 0.01 (**), respectively.

### Data availability.

The whole genome and other related information of phage ϕAFP1 and A. abrolhosensis strain JKT1 were deposited at figshare (https://www.doi.org/10.6084/m9.figshare.21089614). The raw sequence of RNA-seq is available in the SRA database under accession number SRR13374388 (BioProject no. PRJNA689384).
