# Supplementary material for: A Novel Inovirus Reprograms Metabolism and Motility of Marine Alteromonas
Source: Microbiol Spectr. 2022 Oct 27;10(6):e03388-22. doi: 10.1128/spectrum.03388-22 (PMC9769780; doi:10.1128/spectrum.03388-22)
Supplement: Supplemental file 1 — Fig. S1 to S10. Download spectrum.03388-22-s0001.pdf, PDF file, 3.4 MB [file spectrum.03388-22-s0001.pdf]

Supplemental Material figures

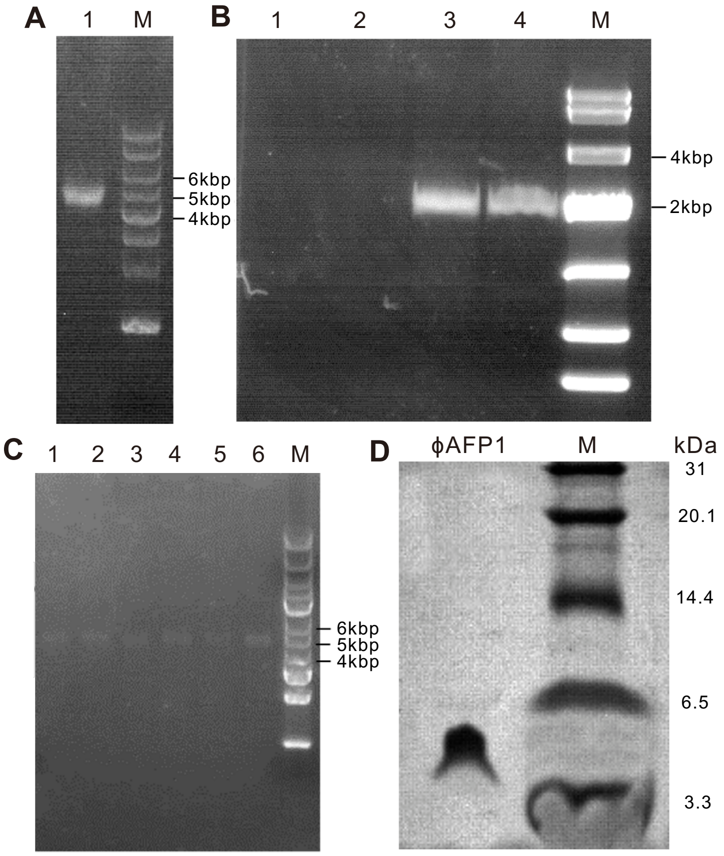

Figure S1 Genomic DNA and protein analysis of phage ϕAFP1. (A) Agarose gel electrophoresis of the genomic DNA of phage ϕAFP1. Lane 1, phage ϕAFP1 genomic DNA. (B) Agarose gel electrophoresis of the genomic DNA of phage ϕAFP1 treated with DNase I (Lane 1), S1 nuclease (Lane 2) or RNase A (Lane 3). Lane 4: untreated ϕAFP1 genomic DNA. (C) Agarose gel electrophoresis of the genomic DNA of ϕAFP1 treated with general restriction enzymes, EcoR Ⅰ(Lane 1), Pst Ⅰ(Lane 2), Xba Ⅰ (Lane 3), BamH Ⅰ(Lane 4), Hind Ⅲ (Lane 5) or untreated (Lane 6). (D) Tris-tricine-

SDS-PAGE gel image of purified phage ϕAFP1 staining with Coomassie brilliant blue R-250. Lane M, 1 kb DNA Marker.

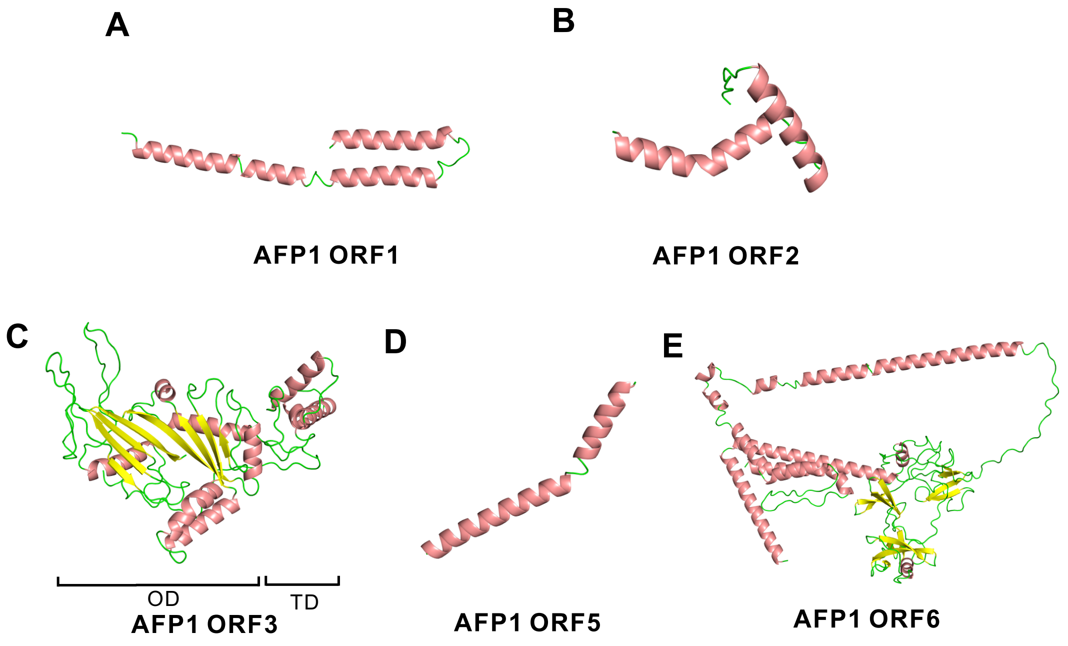

Figure S2 Predicted structures with highest confidence of ORFs proteins from ϕAFP1. ORF1 (A), ORF2 (B), ORF3 (C), ORF5 (D) and ORF6 (E) from ϕAFP1.

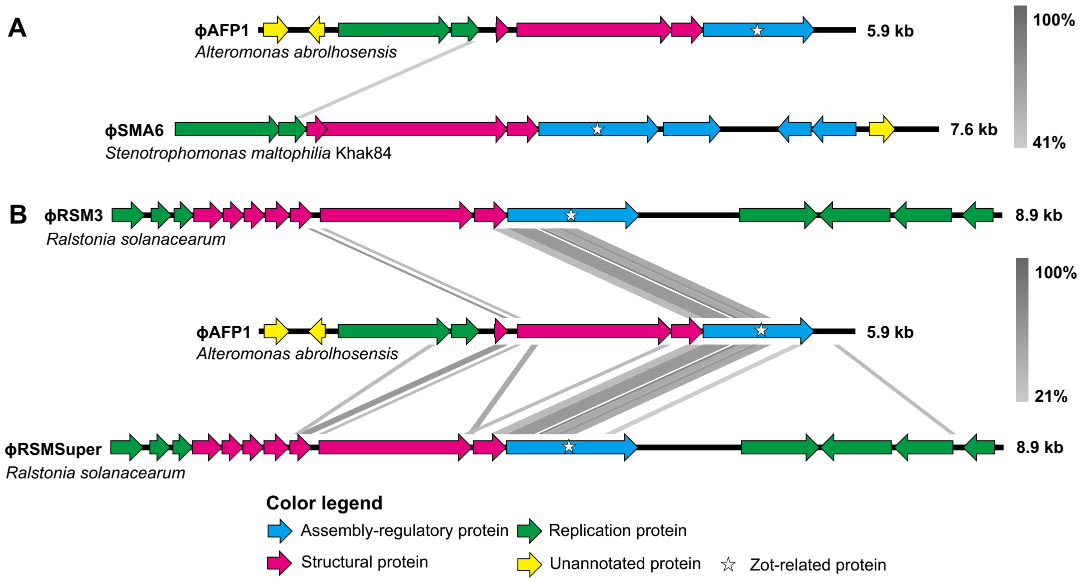

Figure S3 Comparative analysis of the linear genomic organization of ϕAFP1 and other filamentous phages, Stenotrophomonas phage (A) and Ralstonia phage (B). Arrows with different colors represent the putative ORFs belonging to functional modules. Genes belonging to the replication module are marked in green, genes belonging to the structure module are marked in red, genes belonging to the assembly-regulatory module are marked in blue, and unannotated genes are marked in yellow. Further details for comparative analysis were listed in **Supplementary Table 1.**

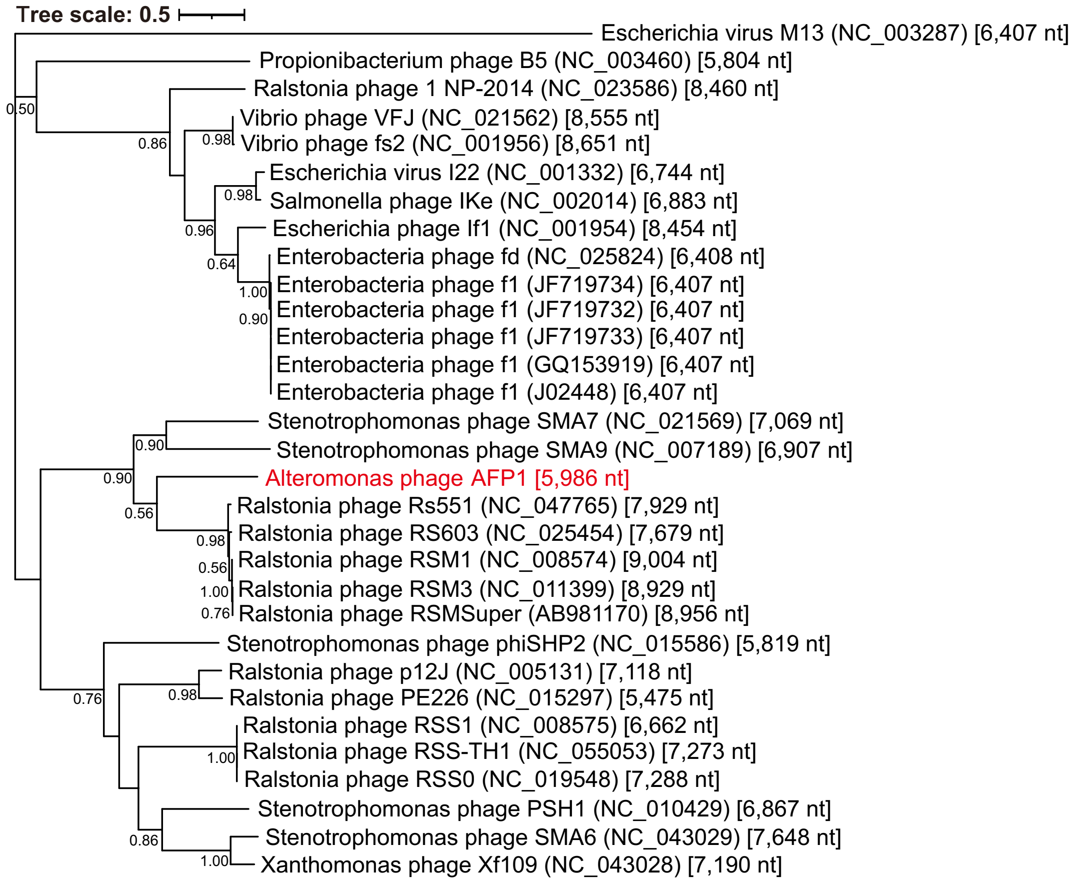

Figure S4 Maximum-likelihood phylogenetic trees generated based on amino acid sequences of Zot-like proteins (pI) of ϕAFP1 and other different filamentous phages. NCBI GenBank accession numbers of phages represented on the tree were listed. The numbers at the nodes represent Bootstrap values and scale bars indicate the average number of substitutions per site. Further details for phages represented on the tree were listed in **Supplementary Table 4**.

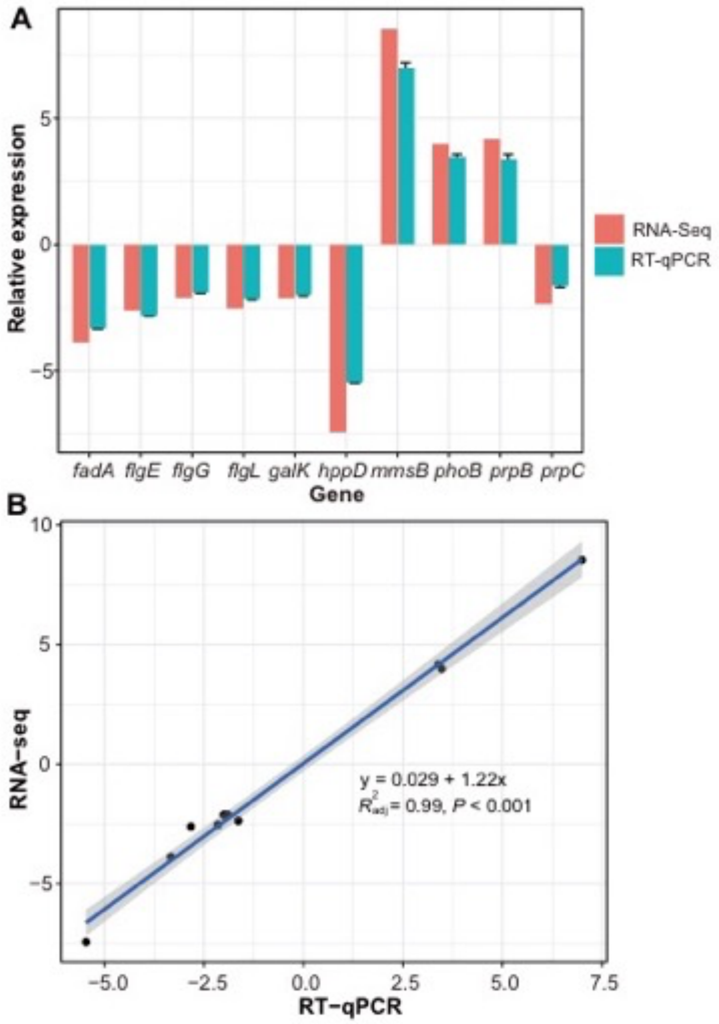

Figure S5 RNA-seq data was verified by RT-qPCR. Comparison of selected DEGs from RNA-Seq and RT-qPCR based on relative expression (A) and Linear regressions (B). Further details for RT-qPCR can be found in **Supplementary Table 10 and 14**.

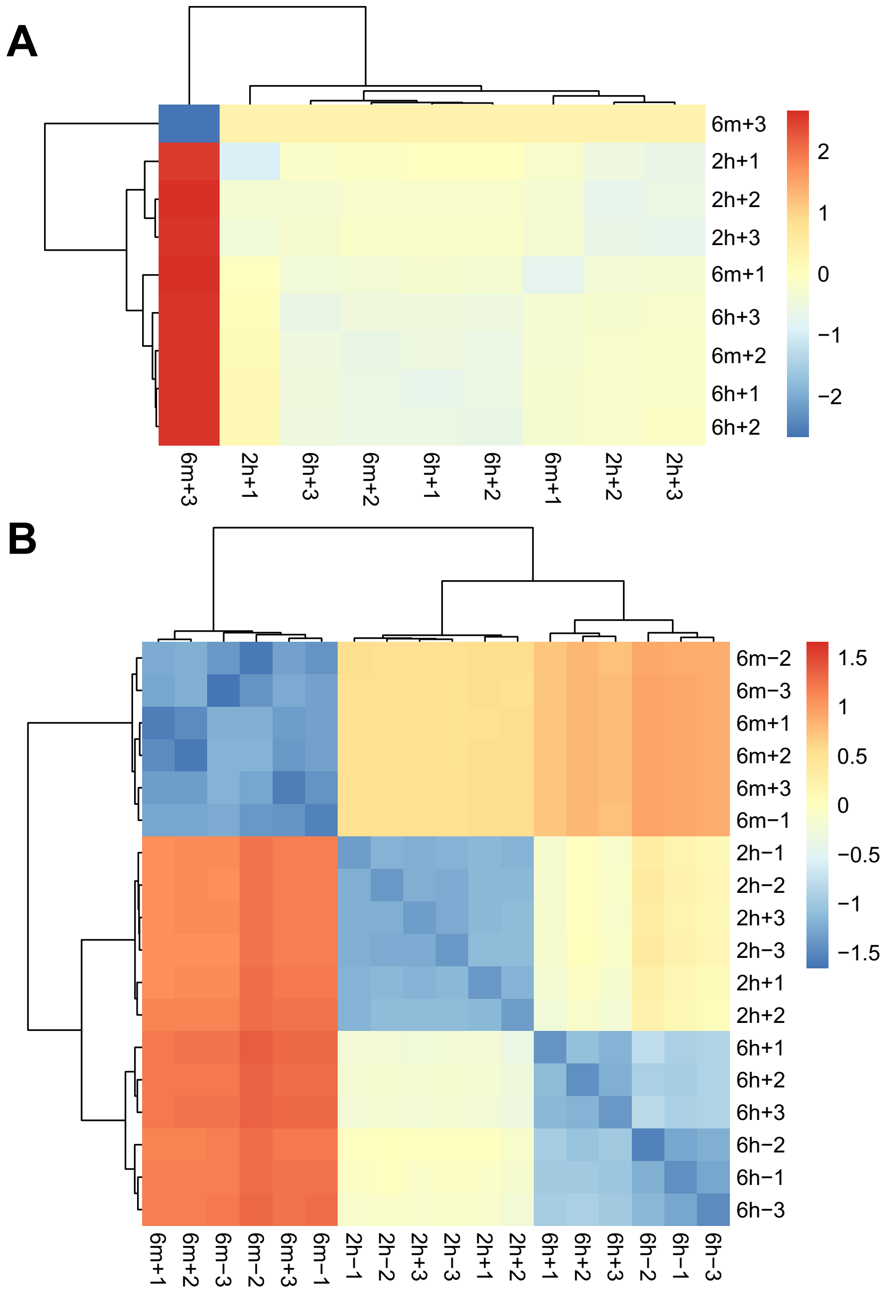

Figure S6 Distance heat map among different samples based on ϕAFP1 genes (A) and *A. abrolhosensis* genes (B) using bray-curtis methods.

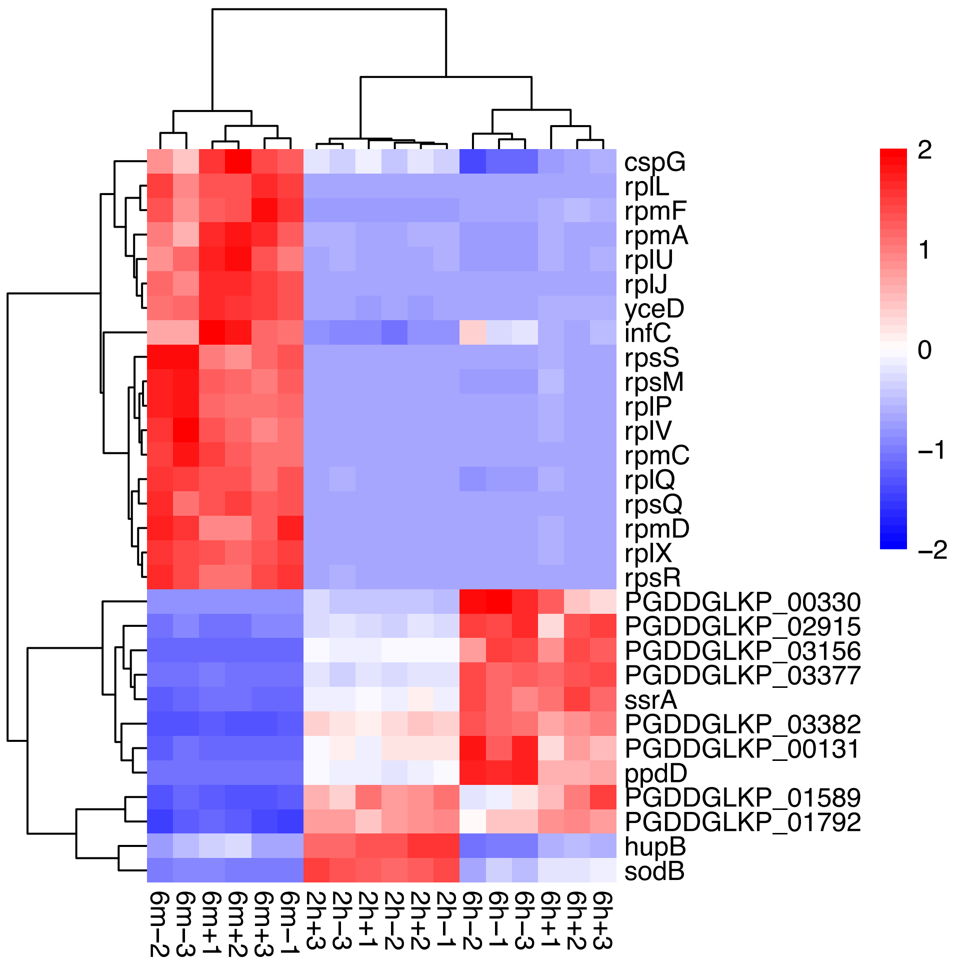

Figure S7 Clustering heat map of the top 30 expressed *A. abrolhosensis* genes among different samples. Further details for expression abundance of host genes can be found in **Supplementary Table 11**.

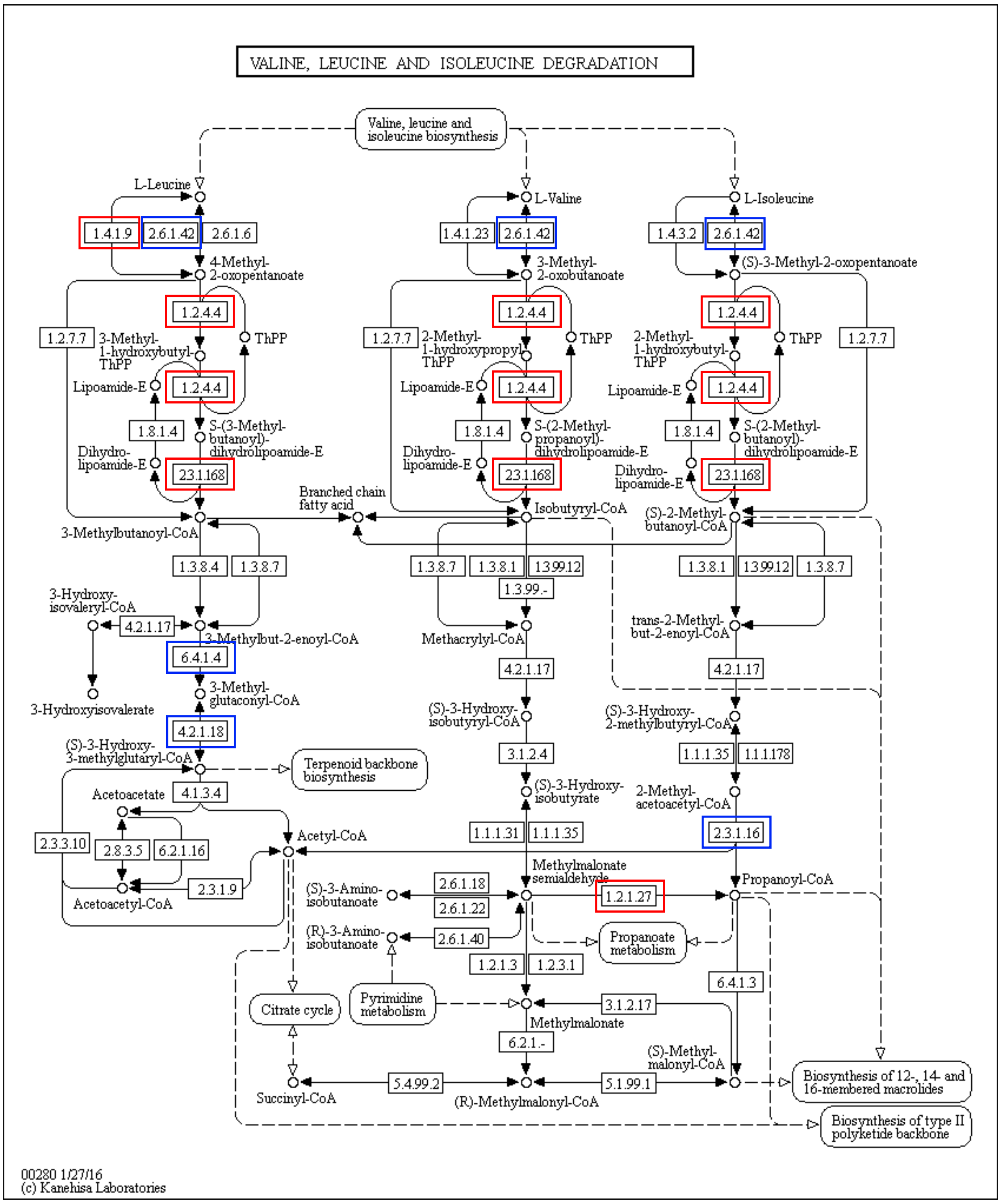

Figure S8 KEGG pathway diagram of valine, leucine and isoleucine degradation (map00280). The red border indicates up-regulation of differentially expressed genes and blue border indicates down-regulation.

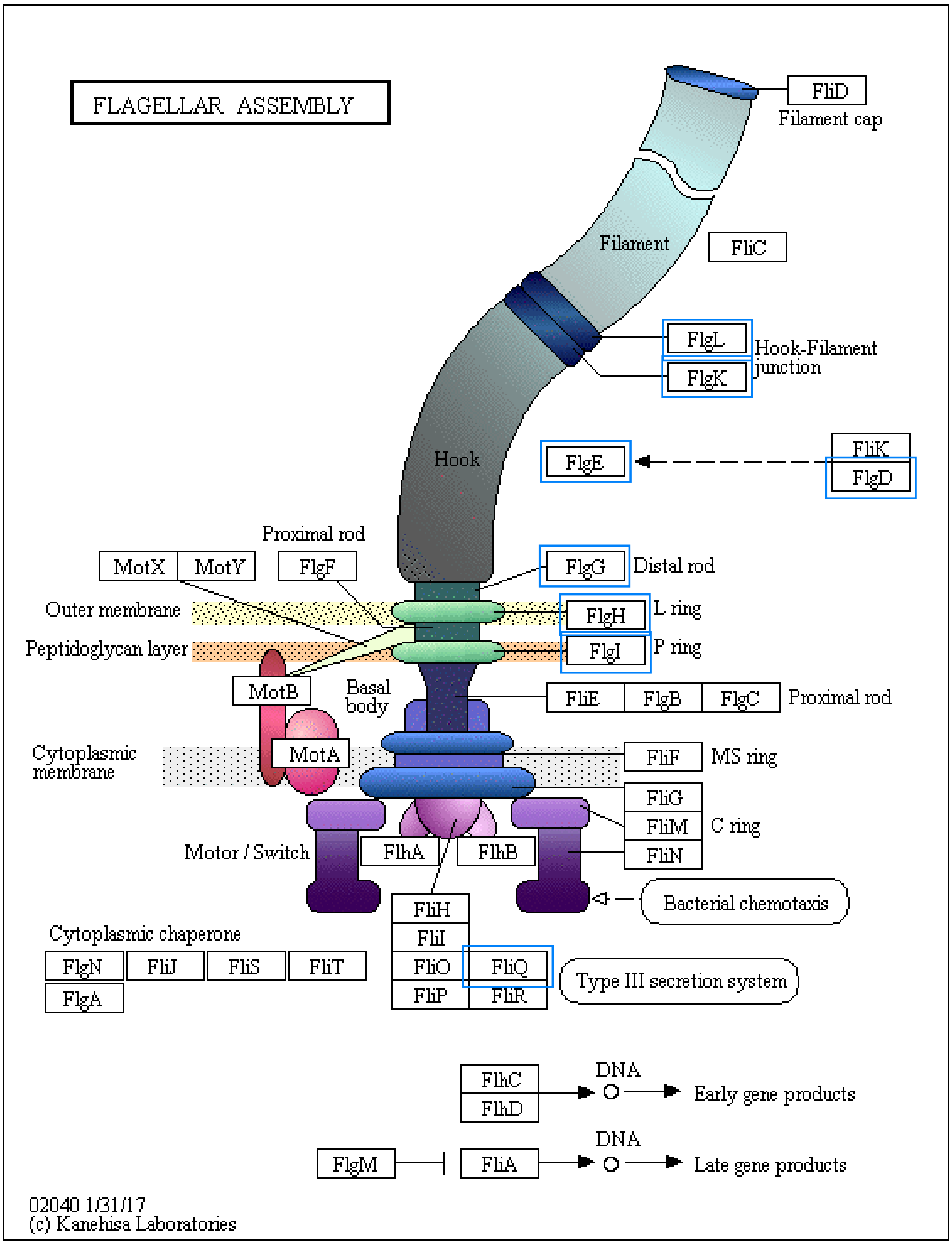

Figure S9 KEGG pathway diagram of flagella assembly (map02040). The blue border indicates down-regulation of differentially expressed genes.

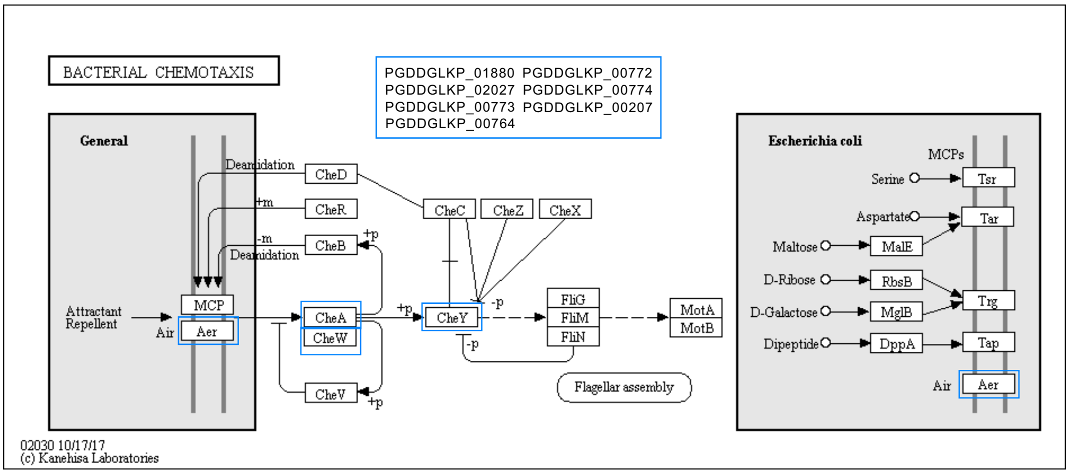

Figure S10 KEGG pathway diagram of bacterial chemotaxis (map02030). The blue border indicates down-regulation of differentially expressed genes and some genes of *A. abrolhosensis* clustered into map02030 are shown in the diagram.
